# Presenilins are required for maintenance of neural stem cells in the developing brain

**DOI:** 10.1186/1750-1326-3-2

**Published:** 2008-01-08

**Authors:** Woo-Young Kim, Jie Shen

**Affiliations:** 1 Center for Neurologic Diseases, Brigham & Women's Hospital, Program in Neuroscience, Harvard Medical School, Boston, MA, 02115, USA; 2 Department of Thoracic/Head and Neck Medical Oncology, The University of Texas M. D. Anderson Cancer Center, Houston, TX, 77030, USA

## Abstract

The early embryonic lethality of mutant mice bearing germ-line deletions of both *presenilin *genes precluded the study of their functions in neural development. We therefore employed the Cre-loxP technology to generate *presenilin *conditional double knockout (*PS *cDKO) mice, in which expression of both presenilins is inactivated in neural progenitor cells (NPC) or neural stem cells and their derivative neurons and glia beginning at embryonic day 11 (E11). In *PS *cDKO mice, dividing NPCs labeled by BrdU are decreased in number beginning at E13.5. By E15.5, fewer than 20% of NPCs remain in *PS *cDKO mice. The depletion of NPCs is accompanied by severe morphological defects and hemorrhages in the *PS *cDKO embryonic brain. Interkinetic nuclear migration of NPCs is also disrupted in *PS *cDKO embryos, as evidenced by displacement of S-phase and M-phase nuclei in the ventricular zone of the telencephalon. Furthermore, the depletion of neural progenitor cells in *PS *cDKO embryos is due to NPCs exiting cell cycle and differentiating into neurons rather than reentering cell cycle between E13.5 and E14.5 following PS inactivation in most NPCs. The length of cell cycle, however, is unchanged in *PS *cDKO embryos. Expression of Notch target genes, *Hes1 *and *Hes5*, is significantly decreased in *PS *cDKO brains, whereas *Dll1 *expression is up-regulated, indicating that Notch signaling is effectively blocked by PS inactivation. These findings demonstrate that presenilins are essential for neural progenitor cells to re-enter cell cycle and thus ensure proper expansion of neural progenitor pool during embryonic neural development.

## Background

During mammalian neural development, neural progenitor cells (NPCs) divide and populate the progenitor pool in the ventricular zone (VZ) of the developing telencephalon [1]. There are two phases of neurogenesis. The first phase spans from embryonic day 8.5 (E8.5) to 10.5, during which neural progenitor population expands. The second phase commences around E11.5, reaches peak by E15.5, and then diminishes by E17.5, generating most of the cortical neurons through eleven cell cycles [2-6]. During the first phase, neural progenitor cells are columnar, connecting the pial surface and the apical surface, and predominantly undergo symmetric cell division to expand rapidly the self-renewal founder progenitor cells [6,7]. During the second phase, neural progenitors in the ventricular zone predominantly undergo asymmetric cell division to generate self-renewal daughter progenitor cells that remain in the cell cycle and committed postmitotic neural precursor daughter cells that will ultimately differentiate into neurons or glia [3,7-10]. Notch receptors are required for neural progenitor cell proliferation [11-13]. In the cell cycle of this asymmetric cell division, at the transition from S phase to M phase, the dividing neural progenitor cells displace their nuclei and subsequently migrate from the basal membrane to the apical surface of the neuroepithelium, a phenomenon termed interkinetic nuclear migration (INM) [14,15].

*Presenilin-1 *(*PS1*) and *presenilin-2 *(*PS2*) are the major causative genes of familiar Alzheimer disease (FAD) [16]. Presenilin (PS) is an essential component of the γ-secretase complex, and Notch is one of the physiological substrates of γ-secretase [17]. Notch is one of the most important regulators of neural development in organisms from insects to mammals [18-20]. During mammalian neural development, activation of the Notch signaling pathway is involved in the maintenance of neural progenitor identity and the decision of cell fate [21]. Presenilin is required for Notch signaling during neural development, though expression of *Hes1*, one of the Notch downstream effector genes, is not suppressed by inactivation of *PS1*, whereas expression of *Hes5 *is effectively suppressed [22]. The germ-line deletion of *PS1 *in mice results in perinatal lethality, cerebral hemorrhages and impairment of neurogenesis, likely due to down-regulation of Notch signaling [22,23]. Although no phenotypes have been observed in *PS2*-null mice [24], earlier and more severe developmental defects are found in *PS1/PS2 *double knockout mice than in *PS1 *single knockout mice [25]. This finding suggests functional redundancy between PS1 and PS2 in embryonic development. When *PS1 *inactivation is restricted to NPC and NPC-derived neurons and glia beginning at ~E11 during the second phase of neurogenesis, the NPC-*PS1 *cKO mice can live up to 2–3 months postnatally. These mice harbor defects in neuronal migration, cortical lamination and radial glial development with milder loss of the progenitor cells [26].

Here we investigate that role of both presenilins in neural development, especially in the maintenance of neural progenitor or stem cell population. We employed a *Nestin-Cre *transgene to circumvent the early embryonic lethality associated with the germ-line deletion of both *PS1 *and *PS2 *loci and to restrict *PS *inactivation to NPCs and NPC-derived neurons and glia. Using this conditional double knockout mouse model, we found that presenilins are required for neural progenitor cells to re-enter cell cycle and for the expansion of neural progenitor population during embryonic development. Precocious exit of cell cycle observed in *PS *cDKO mice is accompanied by disruption of interkinetic nuclear migration and is followed by premature differentiation into postmitotic neurons. Blockade of expression of Notch downstream target genes, *Hes1 *and *Hes5*, in *PS *cDKO mice may underlie or contribute to the failure of neural progenitor cells to re-enter cell cycle. Thus, presenilins play an essential role for NPCs or neural stem cells to retain their self-renewal capacity to expand the progenitor population.

## Results

### Gradual loss of *PS1 *transcripts in the developing *PS *cDKO brain

To determine the spatial and temporal course of *PS1 *inactivation, we performed *in situ *hybridization analysis on the neural progenitor cell (NPC)-restricted *PS *cDKO mice, which were generated by crossing floxed *PS1 *mice with the *Nestin-Cre *transgenic mice in the *PS2*-/- background. We compared the *PS1 *mRNA expression in *PS *cDKO (*fPS1/fPS1; Nestin-Cre; PS2*-/-) embryos and littermate controls (*PS2*-/-) using a probe specific for *PS1 *exons 2 and 3. Consistent with our previous report on the analysis of spatial and temporal inactivation of PS1 in NPC-*PS1 *cKO mice [26], we found a marked reduction of *PS1 *mRNAs in *PS *cDKO embryos at E11.5. At E12.5, no significant signal of *PS1 *mRNAs was detected in the developing telencephalon of *PS *cDKO embryos. By E13.5, the *PS1 *mRNA was no longer detectable throughout the brain (data not shown).

### Severe reduction of neural progenitor cells in *PS *cDKO mice

**Figure 1:**
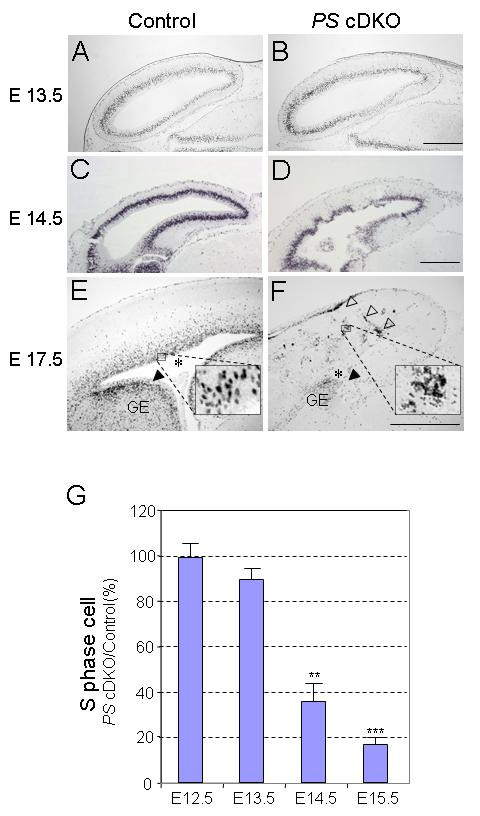
**Depletion of neural progenitor cells in the *PS *cDKO embryonic brain**. (**A-F**) Proliferating (S-pahse) neural progenitors are identified by BrdU labeling at E13.5, E14.5 and E17.5. While the BrdU staining pattern does not differ between *PS *cDKO and control embryonic brains at E13.5 (A, B), severe morphological abnormalities of BrdU stained progenitor cells are present in *PS *cDKO brains at E14.5 (C, D). By E17.5, only a few proliferating neural progenitor cells remain in the lateral ganglionic eminence (GE, black arrowheads), whereas in the ventricular zone of the telencephalon neural progenitor cells are almost completely depleted in the *PS *cDKO mice brain. Many non-neuronal, unnucleated, clotted and small staining are found in the *PS *cDKO brain (opened arrow heads and inset in F). The lateral ventricle (labeled with an asterisk in the control brain) is missing in *PS *cDKO brains (E, F). The insets in E, F are the high power view of boxed area. Scale bar: 500 μm. (**G**) Quantification of BrdU-labeled S-phase cells. BrdU-positive cells were counted at E12.5, E13.5, E14.5, and E15.5, and the percentage of BrdU-positive cells in *PS *cDKO embryos to that in the control is shown. Significant reductions of BrdU-positive cells are detected at E14.5 and E15.5 in *PS *cDKO mice (**, p < 0.01; ***, p < 0.001).

The NPC-restricted *PS *cDKO mice die perinatally. After E15.5, the developing *PS *cDKO brain appeared smaller and grossly abnormal compared with brains from littermate *PS2*-/-mice, in which no abnormal brain phenotype has been reported [27]. At postnatal day 0 (P0), the *PS *cDKO brain is much smaller with lower neuronal density and disorganized cortical layers. To assess whether the number of neural progenitor cells in *PS *cDKO brains was affected, we performed BrdU pulse labeling on comparable brain sections from *PS *cDKO and control mice between E12.5 and E17.5. BrdU is an analogue of thymidine, thus labels newly synthesized DNA. In the control brain, BrdU-positive cells in S-phase reside in the deeper layers of the ventricular zone at these developmental stages [4]. At E13.5 the BrdU staining pattern was not noticeably different between control and *PS *cDKO mice (Figure 1A, B). By E14.5, *PS *cDKO brains exhibit disrupted BrdU staining pattern in many areas and the lateral ventricle was smaller (Figure 1C, D). At E17.5 the lateral ventricle became hardly visible in the *PS *cDKO brain (asterisks in Figure 1E, F), and only a few BrdU-positive progenitor cells remain in the ganglionic eminence (GE) of the mutant cortex (black arrowheads in Figure 1E, F). Severe hemorrhages were also observed in all of the *PS *cDKO brains after E15.5. Many blood-originated, non neuronal staining were found in the mutant brain (open arrowheads in Figure 1F, compare the insets in Figure 1E, F). The number of BrdU-positive progenitor cells in the telencephalon of *PS *cDKO mice was normal at E12.5 (99.3 ± 6.2%) and slightly reduced at E13.5 (89.5 ± 4.8%; Figure 1G). By E14.5 and E15.5, the number of BrdU-positive progenitor cells was further decreased in the *PS *cDKO brains (E14.5: 35.2 ± 7.9%, p < 0.01; E15.5: 17.2 ± 2.5%, p < 0.001; Figure 1G). The results demonstrate that inactivation of presenilins causes drastic reduction of dividing neuroprogenitor cells after E13.5.

### Disrupted interkinetic nuclear migration of neural progenitor cells in *PS *cDKO mice

**Figure 2:**
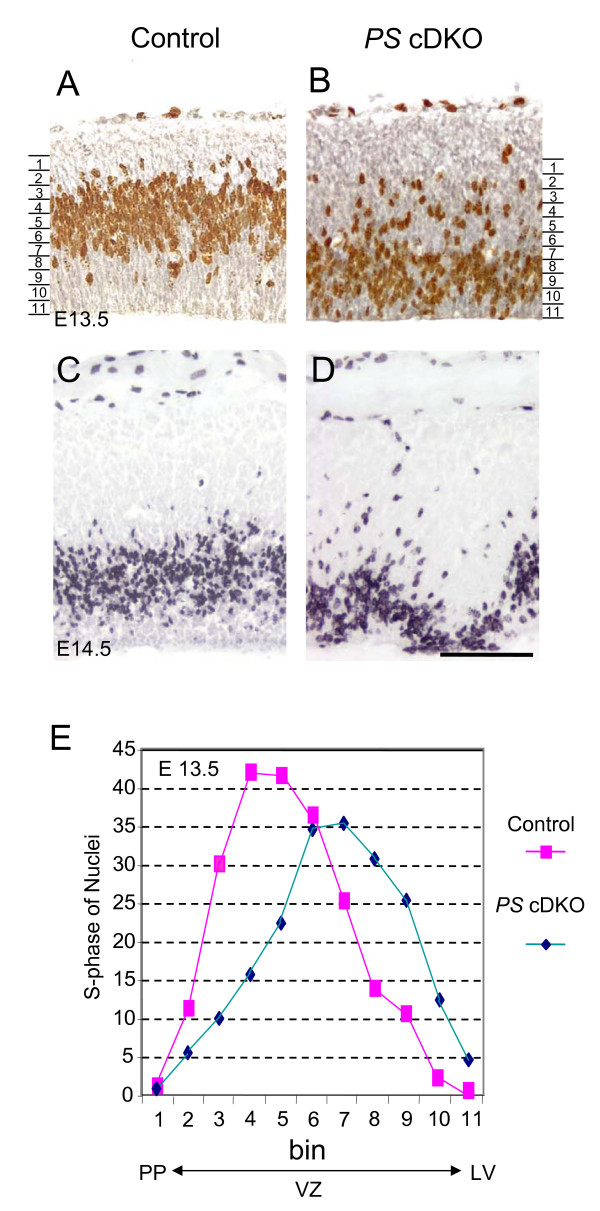
**Ectopic localization of S-phase nuclei in the ventricular zone of *PS *cDKO brains**. (**A, B**) At E13.5, in control brains, the BrdU-positive nuclei are localized in the upper ventricular zone closer to the preplate, whereas in *PS *cDKO brains, they are distributed more broadly and predominantly in the lower ventricular zone closer to the ventricle, suggesting disrupted interkinetic nuclear migration. (**C, D**) At E14.5, in *PS *cDKO brains, the BrdU-positive nuclei are absent in many areas of the ventricular zone and are distributed close to the lateral ventricle. (**E**) The ventricular zone that contains BrdU-positive cells is divided by 11 bins (15 μm/bin). Bin 1 is the layer closest to the preplate (PP), whereas bin 11 is the layer closest to the lateral ventricle (LV). n = 5 for the controls and n = 6 for *PS *cDKO embryos. Scale bar: 100 μm in all figures.

During the cell cycle neural progenitor cells in the developing forebrain undergo a process termed interkinetic nuclear migration (INM) [28]. We then examined whether inactivation of presenilins affects the INM process. At E12.5 and earlier stages, no difference was observed between *PS *cDKO and control brains. By E13.5, however, the S-phase nuclei in the mutant mice were detected in abnormal positions; rather than localized in the basal are of the ventricular zone, they are randomly distributed in the ventricular zone and are especially clustered near the ventricle (Figure 2A, B). To quantify the ectopic distribution of the S-phase nuclei, we divided the cortical ventricular zone into 11 bins from the preplate to the ventricle and then counted the number of the S-phase nuclei in each bin (Figure 2E). The quantitative analysis further confirmed the ectopic localization of the S-phase nuclei at this developmental stage. By E14.5, the interkinetic nuclear migration was further disrupted in *PS *cDKO brains with the S-phase nuclei clustered near the ventricle surface in many places (Figure 2C, D).

**Figure 3:**
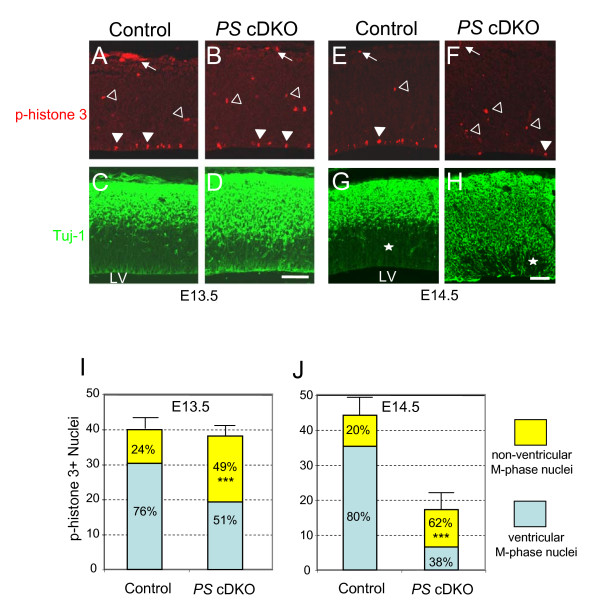
**Displacement of M-phase nuclei in the ventricular zone of *PS *cDKO brains**. (**A-H**) Phospho-histone 3 (p-histone 3, red) is used as a marker for cells in M-phase (A, B, E, F), whereas Tuj-1 (green) is used as an early neuronal marker (C, D, G, H). While the majority of the M-phase nuclei are found in the ventricular surface (closed arrowheads) of the control brain, many M-phase nuclei are found in basal are of the ventricular zone (opened arrowheads) in the developing cortical plate of *PS *cDKO brains at both E13.5 and E14.5, where strong Tuj-1-positive neuronal staining is observed. At E 14.5, most parts of the cortex are stained by Tuj-1 already in the mutant while clear layers are observed in the control. The pan early neuronal marker stained most area of the *PS *cDKO brain and only a very small part of the VZ are still left unstained (white star), suggesting that most of the neural progenitor cells are already being differentiated in the *PS *cDKO brain. The arrows indicate the meningeal cells. Scale bar: 100 μm. (**I, J**) Number and the location of p-histone 3 staining in the parasagital section of forebrain (750 μm width). At E13.5, in the sections the staining number is not different between the genotype. 76.0% ± 2.1% (SEM) of M-phase nuclei are detected in the VS (ventricular M phase nuclei) and about 24.0% of the M-phase nuclei were found in basal are of ventricular zone (non-ventricular M phase nuclei) in the control brain. 49.4% ± 3.6% of the M-phase staining in the *PS *cDKO brain are observed in the non-ventricular region, suggesting that the interkinetic nuclear migration is disrupted in the *PS *cDKO embryonic brain. n = 4 for each genotype (**I**). At E14.5, the p-histone 3 staining is much less in the *PS *cDKO embryos than control (p < 0.05). 20.1% ± 2.4% of the M-phase nuclei are detected in non ventricular position in the control, whereas 61.6% ± 4.9% of those are in non ventricular position in the *PS *cDKO ; n = 6 and 4 for the wild and mutant embryos respectively (**J**). (***, p < 0.001).

We further performed additional immunostaining to determine the localization of the progenitor cell nuclei in M-phase of the cell cycle using phospho-histone 3 (p-histone 3) as a marker. At E13.5, in the control embryo cortex, the p-histone 3-positive nuclei were largely restricted to the ventricular surface (VS), as expected. However, the number of p-histone 3-positive nuclei localized at the basal are of ventricular zone (non-ventricular M-phase nuclei) was significantly higher in *PS *cDKO mice (Figure 3A, B and 3I, 24.0 ± 2.1% for control and 49.4 ± 3.6% for *PS *cDKO, p < 0.001). However, the total number of M-phase cells was unchanged at this time (39.7 ± 4.2 for control, 37.8 ± 3.1 for *PS *cDKO), similarly to S-phase cells we observed (Figure 1). The Tuj-1 staining showed a broader staining pattern in *PS *cDKO mice, indicative of excessive neuronal differentiation (Figure 3C and 3D). In *PS *cDKO mice, the p-histone 3-positive nuclei were found in the Tuj-1-positive zone, suggesting precocious differentiation of the progenitor cells to neurons and the disruption of the boundary between the ventricular zone and the preplate. This genotypic difference became more profound in embryos at E14.5, when much fewer p-histone 3-positive nuclei were present in *PS *cDKO (17.3 ± 2.7) embryos than in the control (44.2 ± 7.9, p < 0.5). Furthermore, a higher percentage of the p-histone 3-positive nuclei were localized at the basal are of ventricular zone in *PS *cDKO mice (61.6 ± 4.9%), compared to the control (20.1 ± 2.4%, p < 0.001; Figure 3E, F, and 3J). The Tuj-1-positive cells cover most of the ventricular zone at this stage in *PS *cDKO mice (Figure 3G and 3H). These results demonstrate the disruption of interkinetic nuclear migration of neural progenitor cells in *PS *cDKO mice.

### Premature exit of the cell cycle in neural progenitor cells of *PS *cDKO mice

**Figure 4:**
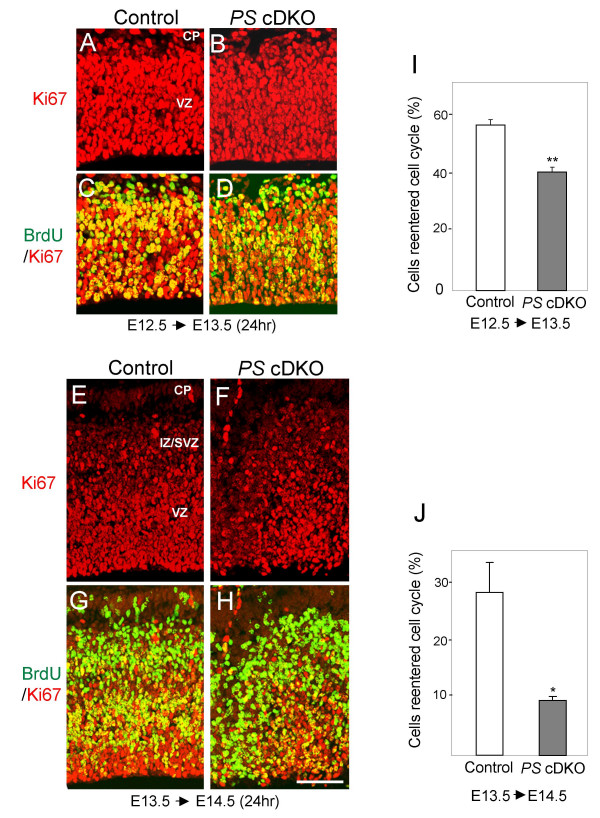
**Reduced percentage of neural progenitor cells reentering the cell cycle in *PS *cDKO embryos**. Immunofluorescent staining of BrdU (green) and the proliferating cell marker Ki67 (red, staining all cell cycle stages except G_0_). (**A-D**) E13.5 embryos labeled by BrdU at E12.5. (**E-H**) E14.5 embryos labeled by BrdU at E13.5. (**I, J**) The proportions of cells which reentered cell cycle are shown as (%). Much less portion of the neural progenitor cells reentered the cell cycle in the *PS *cDKO embryonic brain (*, p < 0.05). Scale bar: 100 μm.

To determine whether the decrease in the progenitor cells and the disruption of INM result from the progenitors exiting the cell cycle to differentiate rather than re-entering the cell cycle, we examined the cell cycle exit and re-entry by calculating the fraction of the cells remaining in the cell cycle during the 24-hour time span. BrdU was used to label dividing progenitor cells at the time of injection, and Ki67 immunoreactivity was used to mark cells that remain in the cell cycle 24 hours later. Thus, dividing cells labeled by BrdU that have left the cell cycle are BrdU+/Ki67- (green), whereas dividing cells that remain in the cell cycle are BrdU+/Ki67+ (yellow in Figure 4A–H). At E13.5, the percentage of the progenitor cells remaining in the cell cycle 24 hours after the BrdU injection at E12.5 is lower in *PS *cDKO mice (40.2% ± 1.6%), compared to that in the control (56.2% ± 1.9%, p < 0.01; Figure 4I). At E14.5, the percentage of the cells remaining in the cell cycle 24 hours after BrdU injection is further decreased in *PS *cDKO mice (9.8% ± 0.5%), relative to the control (28.4% ± 5.1%, p < 0.05) (Figure 4J). Therefore, in the absence of PS, most progenitor cells exit the cell cycle and differentiate into neurons rather than remain as dividing progenitor cells, resulting in depletion of progenitor cells in *PS *cDKO mice.

**Figure 5:**
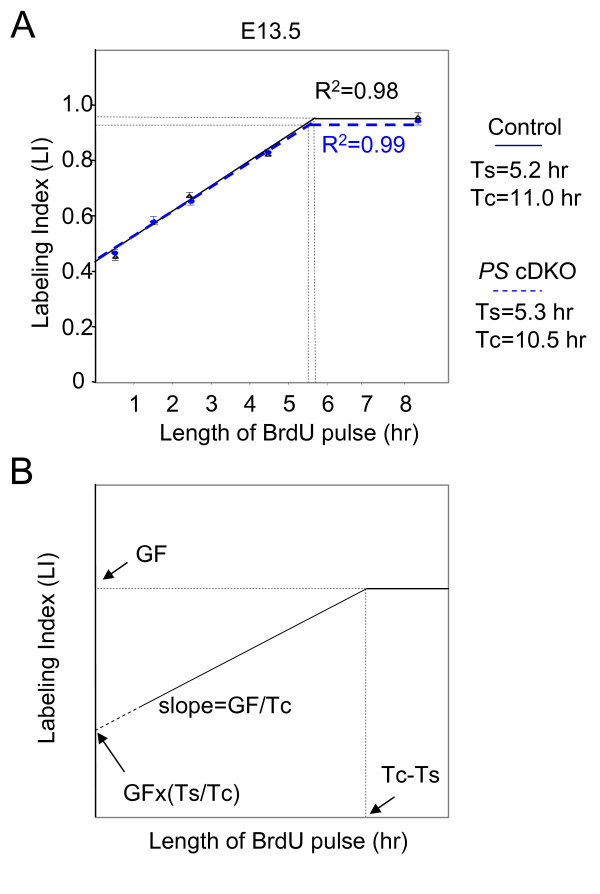
**Unchanged cell cycle kinetics in the *PS *cDKO embryonic brain**. (**A**) Graphs show results of cumulative labeling with BrdU at E13.5 in *PS *cDKO (blue dotted line) and control embryos (black line). Least squares fit analysis of these rising phases (excluding data after giving 8.5 h of BrdU to embryos of both genotypes) yielded the trendlines and R^2 ^values shown. Data points are means ± SEM of the proportions of BrdU-labeled cells, labeling index (LI), in the cortex of each embryo. (**B**) The growth fraction (GF), the length of S phase (Ts) and the length of the cell cycle (Tc) are calculated using the following equations. The intercept on the *y*-axis, *Y*i = GF (Ts/Tc); the time where the GF is maximal (Tm) = Tc-Ts ; the rate of increase (i.e. the slope) of the proportion of labeled cells = GF/Tc.

The result that greater proportion of progenitor cells at E13.5 and E14.5 fail to reenter cell cycle and differentiate into postmitotic neurons in *PS *cDKO mice prompted us to examine whether the length of the cell cycle in the mutant is changed, as the longer cell cycle could complicate the interpretation of the results. We investigated the cell cycle kinetics of the embryos at E13.5, when the neural progenitor cell number is not changed significantly in *PS *cDKO mice. Pregnant mice were given repeated injections of BrdU to label cumulatively the proliferating population. The proportions of BrdU-labeled cells in the proliferating zone after different lengths of exposure to BrdU were plotted in Figure 5A. Using the model described by Nowakowski et al. [29] and used by others [4,5,14], these data can be interpreted as summarized in Figure 5B to give the proportion of cells in the proliferate zone that are actually proliferating (i.e. the growth fraction, GF), the length of the cell cycle (Tc) and the length of S phase (Ts). Previously it was reported that the length of the entire cell cycle and the length of S-phase are longer in *PS1-*deficient neural precursor cells than in wild-type controls at E14.5 [30]. However, we found that Tc (10.5) and Ts (5.3) in *PS *cDKO mice were very similar to those of control embryos (11.0 and 5.2, respectively). The Tc and Ts values we obtained in control mice were similar to those reported previously at this age [31]. Therefore we conclude that the cell cycle kinetics was not changed in *PS *cDKO neural progenitor cells.

### Suppression of Notch signaling in the *PS *cDKO embryonic brain

**Figure 6:**
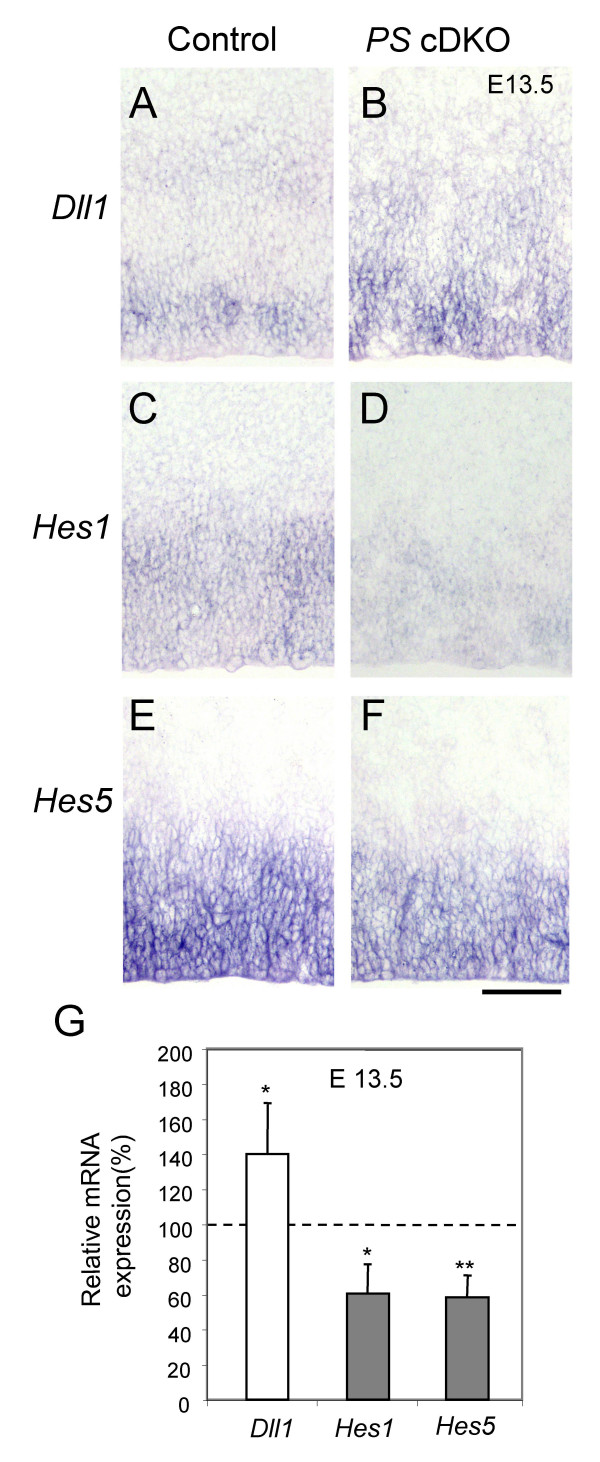
**Suppressed Notch signal in the *PS *cDKO embryonic brain**. The expressions of a notch ligand *Dll1 *and two notch downstream target genes, *Hes1 *and *Hes5*, were examined by *in situ* hybridization (**A-F**) and quantitative RT-PCR (**G**). The comparable E13.5 sections from the control and the *PS *cDKO brain were used. In all parts of the brain, the changes in the expression pattern were similar; the images in the lateral telencephalon are shown. (A, B) The *Dll1 *expression was increased in the *PS *cDKO. (C, D) *Hes1 *was expressed weakly in the control but decreased in the *PS *cDKO. (E, F) The *Hes5 *expression was strong in the control but decreased in the *PS *cDKO. (G) The expressions of the three genes were examined using real-time quantitative RT-PCR. n = 5 for each genotype. All RT-PCR results were concordant with the *in situ* hybridization results (*, p < 0.05; **, p < 0.01). Scale bar: 100 μm.

We further investigated how Notch signaling is affected in the absence of presenilins. *In situ *hybridization analysis showed that expression of one of the Notch ligands, *Dll1*, was increased in *PS *cDKO mice (Figure 6A, B), similarly as what was observed in *PS1*-null mice [22]. Furthermore, expression of both *Hes1 *and *Hes5 *was decreased in the *PS *cDKO embryonic brain (Figure 6C–F). To quantify the differences, we also performed quantitative real-time RT-PCR (Figure 6G). The result showed levels of *Dll1 *(140.1 ± 17.5%, p < 0.05), *Hes1 *(60.6 ± 16.8%, p < 0.01), and *Hes5 *(58.5 ± 9.7%, p < 0.05) transcripts were changed in the *PS *cDKO cortex at E13.5, relative to the control (100%). Thus, Notch signaling in the developing embryonic brain is more effectively suppressed by inactivation of both *PS1 *and *PS2 *in neural progenitor cells than in *PS1*-null mice [22]. The unchanged expression of *Hes1 *in *PS1*-null mice is likely due to the normal expression of PS2, which may compensate for the absence of PS1 on the expression of *Hes1 *during neural development.

## Discussion

The role of presenilins in the second wave of neurogenesis was not studied previously. The regulation of the interkinetic nuclear migration and asymmetric cell division of the neural progenitor cells in the neocortical development was unclear. Here we demonstrate that neural progenitor cells lacking both presenilins fail to maintain their stem cell identity and self-renewal capacity, resulting in depletion of the progenitor population. The interkinetic nuclear migration of neural progenitor cells is also disrupted in the absence of presenilins and is accompanied by the failure of reentering the cell cycle, which leads to precocious neuronal differentiation. Notch signaling was effectively suppressed by inactivation of both presenilins. Together, these findings have uncovered a critical role of presenilins in the cell cycle regulation of neural progenitor cells and the proper expansion of the progenitor population.

### Presenilins in the maintenance of neural progenitor cells through the cell cycle regulation

Germ-line deletion of the *PS1 *gene results in precocious neuronal differentiation and the loss of the neural progenitor cells as early as E10.5 [22]. Conditional inactivation of PS1 in neural progenitor cells of NPC-*PS1 *cKO mice resulted in milder depletion of the progenitor cells, perhaps due to the later onset of PS1 inactivation in *PS1 *cKO mice compared to *PS1*-/- mice [26]. In the current study, we circumvented the early embryonic lethality (~E9) exhibited by *PS1/PS2*-/- mice and restricted inactivation of both PS1 and PS2 to NPCs and NPC-derived cells. Neural progenitor cells in NPC-*PS *cDKO mice are depleted quickly between E12.5 and E15.5, a duration of about 6.5 cell cycles (the length of the total cell cycle at E13 is about 11 hours, see Figure 5), to only 17.2% of the control (Figure 1G).

Despite the fact that interkinetic nuclear migration has been a hallmark of vertebrate neural progenitors for seven decades [32], the mechanism underlying INM is not well understood. Currently, little is known about the molecular mechanisms controlling this unique mode of nuclear movement. Here we found that presenilins are required for the regulation of interkinetic nuclear migration (Figures 2 and 3). It was already severely disrupted in *PS *cDKO mice at E13.5, when the total number of neural progenitor cells was not yet altered. The abnormal location of M- and S-phase nuclei was also accompanied by the failure of neural progenitor cells reentering the cell cycle and the precocious neuronal differentiation (Figures 3 and 4). Therefore, we conclude that presenilins are required for the control of the INM and for the maintenance of daughter cells in the cell cycle, and that the failure of these processes leads to the depletion of self-renewable progenitor cell population.

### Mechanisms of the cell cycle regulation by presenilins

Presenilin may regulate the cell cycle through its downregulation of β-catenin in a γ-secretase-independent manner, as previously reported [33,34]. The transgenic overexpression of β-catenin in neural progenitor cells promoted these cells to reenter cell cycle, leading to the expansion of NPC population and the enlargement of the developing brain [35]. If presenilins negatively regulate β-catenin in the developing brain, the elimination of PS in this study may be expected to activate the Wnt signaling by releasing free β-catenin, a scenario similar to the transgenic overexpression of β-catenin in the brain [35]. However, NPCs lacking PS exit the cell cycle rather than reenter the cell cycle, resulting in depletion of progenitor cells. In addition, we saw no spinal cord abnormality in our NPC-*PS *cDKO mice (WYK and JS, unpublished data), whereas a previous report found elevated nuclear β-catenin in *PS1*-null mice and enhanced cell division in the spinal cord [34]. Therefore, at least for the developmental stages we have studied, the cell cycle dysregulation in the *PS *cDKO mice is unlikely through β-catenin.

A large number of substrates for presenilin-mediated γ-secretase activity have been reported [36]. Loss of the enzymatic activity may contribute the dysregulation of cell cycle in the *PS *cDKO mice Notch is one of the physiological substrates of γ-secretase and is required for the proliferation and survival of NPCs or neural stem cells [11,13,37,38]. The severe depletion of neural progenitor cells in NPC-*PS *cDKO mice in the current study is consistent with the notion that Notch signaling is important for maintaining the NPC population. *Hes5 *expression is reduced in *Notch1*-null mutants and *PS1*-null mice, whereas *Hes1 *expression is not affected in these mutant mice [12,13,22]. Nevertheless, Hes1 is a primary Notch/CBF1 target [39,40] and promotes the cell cycle by suppressing p27^Kip^, a CDK inhibitor [41]. Expression of both *Hes1 *and *Hes5 *is significantly decreased in *PS *cDKO mice, suggesting that the presence of either PS1 or PS2 is sufficient for *Hes1 *expression. When *Hes1 *and *Hes5 *were both mutated, complete loss of neural stem cells was observed [42]. This suggests that the more efficient blocking of *Hes1 *and *Hes5 *expression in *PS *cDKO mice may be the main cause underlying the severe depletion of NPCs. However, substantial levels of *Hes1 *and *Hes5 *were still observed in *PS *cDKO mice, suggesting the existence of presenilin-independent mechanisms for the regulation of Notch signaling [43,44] or Notch-independent regulation of Hes1 expression [45,46].

Notch signaling is important for the regulation of symmetric versus asymmetric cell division of neural stem cells [47,48]. The precise control of this symmetric/asymmetric cell division is essential not only for the nervous system to reach its mature size but also for its proper composition [10,49-51]. Recent reports have shown that two negative modulators of Notch signaling, Numb and Numblike, are important for the maintenance of neural progenitor cells in both earlier stages [52] and later stages [53,54] of neurogenesis in mouse forebrain development. The function of Numb/Numblike in neural progenitor cells is still unclear, because two very similar studies reported opposite results for NPC maintenance; one found more NPCs [53], whereas the other found fewer NPCs [54]. The conclusion of our current study that PS and perhaps Notch signal is required for NPCs to remain in the cell cycle is consistent with the result of the report by Li et al. [53]. Because the *numb/numblike *mutant mice also showed depletion of neural progenitor cells at early stages of neurogenesis [52], it is not yet clear regarding the function of Numb/Numblike in the maintenance of NPC population. Further detailed study is warranted to clarify the functional relation of these molecules in NPC maintenance.

### Presenilins in brain hemorrhages

One of the surprising phenotypes of *PS *cDKO mice is the severe hemorrhage in the entire brain. In *PS *cDKO embryos, hemorrhages are first seen at E14.5 in small numbers of the embryos and by E15.5 spread to the entire brain in all embryos. Nakajima et al. [55] suggested a new function of PS1 in the endothelial cell of blood vessels based on the study using *PS1 *null mice. In the NPC-restricted *PS *cDKO mice, *PS1 *is apparently not eliminated in the mesoderm-derived blood vessel endothelial cells [26,56]. Furthermore, cerebral hemorrhages were not found in NPC-restricted *PS1 *cKO embryonic brains [53]. These findings argue against the cause of the hemorrhage in *PS *cDKO being the possible leakage of the *Nestin-Cre *transgene expression in endothelial cells. The generation of all cell types in the cortex occurs in temporally distinct, albeit overlapping, phases – neurons are generated first (peaking at E14 and finishing perinatally), followed by astrocytes (starting at E13, peaking at P1, finishing by P10), and then oligodendrocytes (starting at E17 and continuing until adulthood) [57,58]. Owing to the severe and almost complete depletion of neural progenitor cells between E12.5 and E15.5, the *PS *cDKO brain may not generate enough astrocytes. Because astrocytes regulate formation of the blood-brain barrier through direct interaction with endothelial cells or by secreting the growth factors, the absence of astrocytes may result in defects in the formation of the blood-brain barrier [59-62]. The related mutant mice (*numb/numblike *cDKO and *PS*1 cKO) that harbor later and milder depletion of neural progenitor cells have not shown cerebral hemorrhages. This suggests that the early depletion of the neural progenitor cells followed by the loss of astrocytes may be the cause of the hemorrhage in the *PS *cDKO brain.

## Methods

### Mice

The generation of neural specific *PS1 *cKO mice using *Nestin-Cre *transgenic and floxed *PS1 *mice [63,64] was described previously [26]. NPC-*PS *cDKO mice were generated by crossing the NPC-*PS1 *cKO mice to the *PS2*-/- background. Specifically, NPC-*PS *cDKO (*fPS1*^Δ^*/fPS1;Nestin-Cre;PS2-/-*, *fPS1*^Δ ^represents a germ-line deletion of the floxed *PS1 *locus) mice used in the study were generated by crossing male *fPS1*^Δ^*/+;Nestin-Cre;PS2-/- *mice with female *fPS1/fPS1;PS2-/-*. Since the floxed *PS1 *locus is linked to the *Nestin-Cre *transgene on the same chromosome, we were able to obtain NPC-*PS *cDKO (*fPS1*^Δ^*/fPS1;Nestin-Cre;PS2-/-*) and littermate control (*fPS1/+; PS2-/-*) embryos at equal ratio. Animal breeding and experiment protocols were in accordance with the National Institute of Health Guidelines for use of live animals and approved by the Harvard Medical Area (HMA) Standing Committee of Animals.

### Preparation of embryos and brain sections

After embryos were fixed in 4% paraformaldehyde at 4°C for 1.5 h (E11.5) or 2 h (E12.5 and later stages), they were cryoprotected and embedded in OCT (Sakura Fine Technical) for frozen sections or dehydrated and embedded in paraffin for paraffin sections. For the S-phase labeling, the pregnant mice were injected intraperitoneally with BrdU/PBS (70 mg/kg body weight; Sigma) 30 min before the dissection. For the cell cycle exit experiments, BrdU was injected into the pregnant mice 24 h before the dissection.

### Immunostaining and in situ hybridization

For immunostainings, sections were blocked with a blocking solution containing 1% BSA, 3% goat serum, and 0.1% Triton X-100 for 3 h at room temperature and incubated with the desired primary antibodies overnight at room temperature. The following primary antisera or antibodies were used: anti-TUJ-1 antibody (1:400, Babco), anti-phospho histone 3 (1:100, Santa Cruz Biotechnology). For immunohistochemistry, the secondary antibody labeled with biotin (1:400, Vector Labs) was incubated, and the ABC complex (Vector Labs) incubation followed. The staining was performed using the DAPI kit (Vector Labs) or AP Purple (Roche). For the fluorescence staining of the tissues, the primary antibody-treated sections were incubated with the appropriate Alexi Fluor 488- or Alexi Fluor 594-conjugated secondary antibodies (1:300, Molecular Probes) for 2 h at room temperature. Images were viewed on a Zeiss confocal laser scanning microscope and analyzed by using LSM 510 software. In situ hybridization of brain sections was performed as described previously [22] using the digoxygenin-labeled riboprobes. The *Hes1*, *Hes5*, and *Dll1 *riboprobes were described previously [22]. All statistical analyses of the counted cells were performed using Microsoft Excel software.

### Interkinetic nuclear migration and cell cycle reentering assay

For S-Phase nuclei staining, the BrdU injected pregnant mice were dissected and the embryos were harvested at E13.5 or E14.5. The frozen sections were stained with an anti-BrdU antibody for S-Phase labeling. The pictures of stained slides were taken and the VZ were divided into 11 bins. The S-phase nuclei in each bin with a width of 500 μm were counted, and the numbers of the nuclei and their positions were shown in the diagram. The M-phase nuclei, which were stained with an anti-phospho-histone 3 antibody, were counted in dorsolateral corte x sections with a width of 750 μm. The portion of cells that re-enter cell cycle were calculated as following: The number of cells labeled with both BrdU and Ki67 (yellow, re-entered cell cycle) was divided by the total number of BrdU stained cells (green, BrdU+/Ki67- and yellow, BrdU+/Ki67+) 24 h after BrdU pulse labeling.

### Cell cycle parameters calculation

Analysis of cell cycle kinetics in the cortex using cumulative BrdU-labeling procedures were performed as described previously [5] with slight modification. Briefly, BrdU pulses (70 mg/kg body weight) were given to pregnant dams every 2 h for up to 8 h. Under these conditions, all nuclei passing through the S-phase will be labeled. Dams at the age of E13.5 were sacrificed 0.5, 1.5, 2.5, 4.5 and 8.5 h after the first injection. The embryos were fixed for 2 h in 4% paraformaldehyde, paraffin-embedded and processed to reveal BrdU immunoreactivity. The proportions of cells in the proliferative zone that were BrdU labeled were counted in 400 μm wide strips throughout the telencephalon.

Labeling index (LI; labeled cells as a proportion of total cells) was calculated for the ventricular zone after the sections were stained with an anti BrdU antibody. After the staining, the labeling index at each time point was calculated as the number of BrdU-positive nuclei divided by the total nuclei count in the ventricular zone. The resulting data were then used to determine the time of BrdU labeling needed to reach the maximum value of BrdU-stained nuclei, which corresponds to the total cell cycle length subtracted by the length of S phase (Tc-Ts).

### Quantitative real-time RT-PCR

At E13.5, cortexes were collected for the preparation of total RNA using Tri reagent (Sigma). The cDNA was synthesized using the Superscript II first-strand cDNA synthesis system (Gibco BRL). Oligonucleotide primers were designed using Primer Express software 1.0 (Applied Biosystems). The specificity of primers was determined by gel electrophoresis of PCR products to ensure a single band with predicted size. Quantitative PCR reaction was performed using SYBR Green on an ABI PRISM 7700 Sequence Detection System (Applied Biosystems). Relative quantification of gene expression was performed using the comparative CT method according to manufacturer's recommended protocol (User Bulletin #2, ABI PRISM 7700 Sequence Detection System, Perkin-Elmer). Levels of target mRNA were normalized using 18S rRNA. The change of the expression of a target gene in the *PS *cDKO embryonic brain relative to the littermate control was calculated as: Fold change = 2^-(ΔCT, Tg-ΔCT, control)^.

## Competing interests

The author(s) declare that they have no competing interests.

## Authors' contributions

WYK planned and performed the experiments and wrote the paper, JS supervised the project and wrote the paper. All authors have read and approved the final manuscript.
